# Social support experiences when growing up with a parent with Huntington’s disease

**DOI:** 10.1080/21642850.2022.2104286

**Published:** 2022-07-29

**Authors:** Siri Kjoelaas, Kristin B. Feragen, Tine K. Jensen

**Affiliations:** a Centre for Rare Disorders, Oslo University Hospital HF, Oslo, Norway; b Department of Psychology, University of Oslo, Oslo, Norway; c Norwegian Centre for Violence and Traumatic Stress Studies, Oslo, Norway

**Keywords:** Social support, Huntington’s disease, adversity, stress, young people

## Abstract

**Background:**

Social support is a strong protector factor against the many negative effects stress and adversity in childhood can have on short- and long-term health. However, for young people who are exposed to adversity because their parent suffers from severe neurodegenerative disease, such as Huntington’s disease (HD), support from close caregiving relationships can be compromised. This study aimed to investigate what current and past experiences young people who grow up with a parent with HD have with social support outside the parent–child context.

**Methods:**

A total of 36 semi-structured qualitative interviews with individuals who had current and past experiences growing up with a parent with HD were analysed using thematic analysis.

**Findings:**

Relationships were experienced as supportive when they provided a sense of love, care, or belonging; when they provided coping skills; and when they reduced or alleviated stressors at home. Barriers to receiving and accepting support included their parent’s and others’ lack of acknowledgement and understanding about their situation and the young people’s own need to protect themselves or their family from support they feared could cause harm.

**Conclusion:**

Our findings highlight the many important roles persons other than caregivers can have in helping young people who grow up with the distress and adversity of having a parent with a severe disease, such as HD. The findings suggest that by sustaining positive and adaptive emotions and/ or changing distressing emotions, social support help and can compensate for a lack of support in their caregiving relationships. In order for others to be experienced as supportive, the many barriers this vulnerable group may encounter must be addressed and overcome. Most importantly, support providers must understand how HD affects young people.

## Introduction

Social support is one of the most significant determinants of psychological health (Thompson, Flood, & Goodvin, 2015). For children and adolescents, studies consistently show that social support can protect against the many damaging effects adversity and distress can have on short- and long-term health (Hughes et al., 2017; Thompson et al., 2015). Young people can be exposed to adversity and distress in many situations, one of which is when their parents suffer an illness or injury that disrupts their ability to provide appropriate care for them. However, we continue to lack in-depth knowledge about how young people who grow up with a caregiver with severe disease, such as Huntington’s disease (HD), are helped by their relationships in coping with and adjusting to the many stressors they endure, as well as what potentially hinders this vulnerable group from receiving the support they need.

Support can come from any part of a young person’s social network, ranging from informal relationships, such as their parents, extended family members, or peers, to formal contacts, such as teachers or healthcare workers (Taylor, 2011). Theory and research has defined and categorised social support in many ways; however, the literature generally relates to relationships that provide a variety of emotional, informational, or instrumental resources (Thoits, 2011). Emotional support occurs when others provide values, such as love or sympathy; informational support occurs when others provide facts or advice; and instrumental support occurs when others provide behavioural or material assistance. While having these types of supportive relationships is beneficial in any life stage, it is perhaps particularly important in helping to maintain mental and physical health in times of stress and adversity. In times of stress, the relationships we have with others have the power to reduce otherwise harmful psychological reactions and promote positive adjustments by acting as a resource and buffer against the effects of stress (Taylor, 2011). When children and adolescents endure significant adversity and distress, having a robust social support system has been found to be one of the most important factors in minimising the many risks these experiences pose to their short- and long-term health (Hughes et al., 2017; Shonkoff et al., 2012). Consequently, children and adolescents who experience significant ongoing adversity without having the help of important relationships have a higher risk of poor mental health, particularly if they experience this adversity at home (Thompson et al., 2015). A central point for interventions that aims to help families with adversity and distress is, therefore, to enhance existing social support and build new networks in which such children can be supported (Thompson et al., 2015).

Because the idea that social relationships are beneficial is an ingrained part of our understanding of health and well-being, it is perhaps natural to assume that it can easily be implemented for families in which children are exposed to significant adversity and distress and that social support will automatically be beneficial. However, the way in which social support actually assists young people in families with high levels of stress and adversity is, in reality, a much more complex and multifaceted process that is made difficult by a range of factors (Thompson et al., 2015). For instance, there are stressful contexts that may not allow youth to draw on the benefits of social support (Rueger, Malecki, Pyun, Aycock, & Coyle, 2016). Whether support will actually help depends on that person’s individual needs and values (Thompson et al., 2015). Also, when considering support, another complicating factor is the need to distinguish between support that is received and support that is perceived. Received support refers to the actual support that is provided, whereas perceived support reflects the person’s sense or experience that support would be available if needed (Taylor, 2011). Interestingly, while perceived support has repeatedly been associated with better mental and physical health, the results are more mixed for received support, where research report weak and even contradictory results (e.g. Bolger & Amarel, 2007; del-Pino-Casado, Frías-Osuna, Palomino-Moral, Ruzafa-Martínez, & Ramos-Morcillo, 2018; Thoresen, Jensen, Wentzel-Larsen, & Dyb, 2014). However, we lack knowledge that takes into account the many ways social support can help young people who grow up with a parent who suffers from severe disease, as well as the barriers this vulnerable group may face in obtaining the support they need. In the current study, we examine social support experiences in a sample of adolescents and adults who grew up in families with a severe and complex illness, Huntington’s disease (HD).

Huntington’s disease is a progressive neurodegenerative disease with a 50% risk of genetic transmission (McColgan & Tabrizi, 2018). The disease has unquestionably devastating effects on those affected, their families, and the young people whose caregivers are affected in particular. The disease will cause damage to the brain that slowly and gradually affects most functional abilities, and symptoms usually appear between 30 and 50 years of age (McColgan & Tabrizi, 2018), a time in life in which many people have caretaking responsibilities for families and children. The burdens on those affected by HD often last a long time, averaging 17–20 years (McColgan & Tabrizi, 2018), and there is currently no cure and few alternatives available for symptom relief. HD is perhaps most detectable via the disease’s visible symptoms, which include uncontrollable movements and changes in motor function (McColgan & Tabrizi, 2018). However, the disease will also create a range of cognitive, and psychiatric symptoms, including personality changes, aggression or apathy, psychosis, a lack of empathy and insight, and difficulties with social perspective-taking years prior to physical symptoms (Eddy & Rickards, 2015; McColgan & Tabrizi, 2018). Although these symptoms are less visible, they may present the most significant challenges to the ability to appropriately care for children.

There exists a small but significant body of research reflecting how HD can impact the lives of young people. Among the multiple and ongoing stressors they may endure are the high risk of being left with overwhelming responsibilities at home (Kavanaugh, 2014) and being exposed to a range of adverse experiences, such as chronic unpredictability, domestic violence, and suicide (Forrest Keenan, Miedzybrodzka, Van Teijlingen, McKee, & Simpson, 2007; Kjoelaas, Jensen, & Feragen, 2021; van der Meer, van Duijn, Wolterbeek, & Tibben, 2012). In addition, many struggle with the constant worry about their own possibility of one day developing the disease and whether or not to get tested as they approach adulthood. It seems many do not get the support they need during this process (Lewit-Mendes, Lowe, Lewis, Corben, & Delatycki, 2018; Forrest Keenan, McKee, & Miedzybrodzka, 2015; Tillerås, Kjoelaas, Dramstad, Feragen, & von der Lippe, 2020). In response to the many and chronic stressors they face, these young people have often been found to struggle with generally poor psychological well-being, including depression and anxiety (Ciriegio et al., 2020; Lewit-Mendes et al., 2018). However, the experiences of these young people do vary, and while some struggle, others cope more successfully with the challenges of growing up with a parent with HD (Forrest Keenan et al., 2007).

To further understand how young people with a parent with HD could be helped to cope and adapt, research from other broader categories of children at risk may be useful, such as at risk of averse childhood experiences (ACE’s; Kjoelaas et al., 2021; van der Meer et al., 2012) or with young people who grow up serving as a caregiver for their parent with physical or mental illness in general, called ‘young carers’ (Becker, 2000). For this group, one systematic-review shows that taking on caregiving tasks in the family can have both a positive and a negative impact on a young person’s development (Chikhradze, Knecht, & Metzing, 2017). A key factor to cope and adapt for young people in families with HD, those at risk of ACEs, and young carers in general, seems to be the protection and guidance provided by having good systems of social support and strong attachments within relationships (e.g. Forrest Keenan et al., 2007; Pakenham, Chiu, Bursnall, & Cannon, 2007; Kjoelaas et al., 2021). Consequently, past research strongly advocates that this group is in need of social support systems that can help them cope and adapt (e.g. Forrest Keenan et al., 2007; Kavanaugh, 2014; Lewit-Mendes et al., 2018). However, we still know little about the ways in which others outside the parent–child context may help young people in families with HD cope with and adapt to the stressors they endure throughout childhood.

What research has suggested is that, for this group of children and adolescents, finding support from others may also not be an easy task. The close attachments they find in their relationships with their caregivers are an important source of social support for any child or adolescent (Bowlby, 1979). However, in families with HD, the disease often disrupts family systems and can compromise the resources and availability of both caregivers as a source of support for their children (Forrest Keenan et al., 2007; Kjoelaas et al., 2021; Mand et al., 2015; Vamos, Hambridge, Edwards, & Conaghan, 2007; Van der Meer et al., 2006). For instance, as one caregiver gradually loses his or her caregiving and supportive abilities due to the progression of HD, the other caregiver may also become less available due to increasing preoccupations with the tasks of caring for their partner (Mand et al., 2015). It is therefore likely that young people in families with HD must rely more heavily on social support from outside the parent–child relationship for help in coping. However, this particular group has also been found to have a range of unmet needs regarding support from others, including help with complex caregiving tasks, and many experience social isolation, receive little support from peers or adults, and want more emotional support (Kavanaugh, 2014; Kavanaugh, Noh, & Studer, 2015; Forrest Keenan et al., 2007).

Given the major risks on short- and long-term health that can come with the enduring stress and adversity many young people with a parent with HD experience with the addition of having support from their caregivers compromised by the disease, understanding how social support can help their coping and adjustment is important for interventions to provide actual help. What possibly hinders this vulnerable group from obtaining the support they need may also be a particularly important piece of this puzzle. Therefore, this study aimed to provide an in-depth understanding of social support for this group. Specifically, we addressed the research question: What are the current and past experiences of social support outside the parent–child context for young people with a parent with HD?

## Methods

### Sample and setting

As a part of a larger national study with the overall aim of exploring the experiences of growing up in a family affected by HD [Regional Committee for Medical Research Ethics Health region East, Norway, reference number: 2017/1613], we analysed interviews collected in 2018. Anyone in Norway over the age of 12 years who had current or previous experiences of growing up with a parent with HD was invited to participate in the study. Information about the study was distributed verbally and through information sheets in several settings where individuals who had grown up in a family affected by HD could be reached. Information about the study was received directly (e.g. via counsellors or other health care professionals) and indirectly (e.g. via the offspring’s caregivers or peers). The main locations where information was distributed included educational courses for families affected by HD, genetic counselling services at Oslo and Haukeland University Hospitals and St. Olavs Hospital, the Norwegian Association for Huntington’s Disease, and Facebook. The information sheets outlined the study’s purpose, provided information about the interview topics, and included the main researcher’s name and contact information and a consent form that could be returned by mail. In response to the formal and informal invitations, 42 people provided initial written consent to participate via mail or at the time of the interviews. Of these, six people could not be reached when subsequently contacted for an interview. A total of 36 participants (26 females, 10 males) were interviewed and included in the analysis. The participants were adolescents, young adults, and adults reflecting on their current and past experiences (age range = 13–65 years; M_age_ = 36.6 years). Table 1 summarises the participant’s demographic information.

**Table 1.** Participant demographic characteristics (*N* = 36).

Variable		Label	*N*
Age	13–18 years	Teenager	7
	19–35 years	Young adult	10
	36–65 years	Adult	19
Gender	Female		26
	Male		10
Parent with HD	Mother		19
	Father		17
Occupational status	Student		8
	Full-time employment		14
	Part-time employment		3
	No employment		8
	Unknown		3
Marital status	In a relationship/married		18
	Single		12
	Unknown		6
Family status	One or more child		16
	No children		17
	Unknown		3

### Data collection procedures

An interview guide was created based on the relevant literature and feedback from clinical experts and a group of user representatives. The clinical expert references that helped to develop the interview guide included four counsellors from the Centre for Rare disorders at Oslo University Hospital, with extensive experience from working with families with HD in a clinical setting. The user representative group consisted of three individuals with experiences of either growing up with a parent with HD or having children with a partner with HD. Individual semi-structured interviews were conducted, focusing on childhood experiences, family relations, and experiences of support. Table 2 displays interview topics and sample questions. Face-to-face interviews were generally preferred (*n* = 33); however, a few participants preferred telephone interviews (*n* = 3). The interviews were conducted at the Centre for Rare Disorders at Oslo University Hospital, in other locations outside the home, or in the homes of a few participants. The locations of the interviews were based on the preferences of our participants. Because many participants had challenging home lives with partners or caregivers with HD or felt it would be easier to talk in the privacy of our out-patient hospital department, most participants preferred to be interviewed outside their homes. Counsellors from the Centre for Rare Disorders with many years of clinical experience with HD were involved in planning the study, but they were not involved in the data analyses, due to their involvement with potential participants. Five researchers conducted the interviews, and these included one clinical psychologist, three postgraduate psychology students, and two trained health professionals at Oslo University Hospital. The students had no previous experience with HD, whereas the health professionals had previous research experience with the disease. All the interviewers had or received formal training in qualitative methods before conducting the interviews and supervision regarding interview techniques was provided if needed. The interviewers had no previous familiarity with the study participants. The project manager, an experienced licenced clinical psychologist, participated in at least two interviews conducted by novice researchers to ensure the reliability and consistency of all interviewers’ practices.

**Table 2.** Main topics and sample items applied in the semi-structured interview guide.

Interview topic	Sample question
Background information	*What is your motivation to participate in this study?*
	*Please describe the family you grew up in?*
Childhood experience	*Tell me about your childhood?*
	*How did your parents’ disease affect your family?*
	*What was your relationship with your mother and father like?*
	*How has growing up with a parent with HD affected you?*
Disease- and self-disclosure	*What is your experience of disclosing information about HD growing up?*
	*What are your thoughts on how parents should inform their children about HD?*
Resources and support	*What relationships were your sources of support growing up?*
	*How did you/ do you feel about having friends over to visit?*
	*How did relationships help you understand and cope with your situation?*

### Data analysis

The interviews were recorded and transcribed verbatim. We followed the guidance of Braun and Clarke’s reflexive (2006, 2019) thematic analysis. During the first and second steps, all the authors became familiar with the data by reading and re-reading the interview transcripts and noted any text related to support. During this step, we noted that lack of support often was spoken of as barriers to support. That is, the participants experiences of what may have stood in the way of receiving support. In the further analysis we therefore separated experiences of support and barriers in two separate superordinate themes. In the following steps, the first and second authors generated initial codes by isolating phrases, sentences, and paragraphs and generated a list of codes representing every transcript for both superordinate themes separately, i.e. support and barriers to support. The lists of codes were collated to search for themes according to the similarities between them. Themes were chosen for their prevalence in relation to the research question(s). These themes were then reviewed against the data and discussed between the three authors until full agreement was reached; the final themes were then determined and named. Finally, the report was produced.

Reflexivity was emphasised throughout the analysis. Two of the authors (the first and the second) have previous research experience with HD. Knowledge about HD was helpful during the analytic phase. However, this experience also had the potential to create biased interpretations of the interviews. Inspired by the consensual qualitative research model (Hill, Thompson, & Williams, 1997), to enhance the study’s validity and counteract group thinking and researcher bias, the first and second author formed the primary analytic team. The third author, who had no previous experience or knowledge of HD, read most interviews independently and served as a discussant during the analyses. Consensus on topics was obtained after repeated rounds of independent reading, sharing notes, discussion, and, finally, rereading and re-discussing the interviews. Last, all cases were analyzed, checking for consistency and soundness, both case by case and across cases. Some participants were young when their parents developed symptoms of HD, whereas others reported memories from late adolescence. The interviewers asked participants to provide the approximate age at which they experienced these events. Given this study’s focus, only experiences that had occurred in childhood or adolescence were included in the analysis.

In the presentation of the findings, the frequency labels *general*, *typical*, and *variant*, as suggested by Hill et al. (2005), are used to indicate the degree of representativeness across individual cases. The themes that were general in the sense that they applied to all but one participant are referred to in the text as *all participants*. Topics were considered typical if they applied to more than half of the cases and are referred to as *most participants*. Topics were variant if they were represented in less than half of the sample but appeared in more than two cases; they are referred to in the text as *some participants.* Quotes that illustrate themes and subthemes were selected and translated from the original language into English. Participants were given pseudonyms and identifying information has been omitted.

### Ethical considerations

Ethical approval for the study was obtained from the Regional Committee for Medical Research Ethics (Health region South-East, Norway*,* reference number: 2017/1613). Participants were informed about the study, provided written consent, and were informed of their right to withdraw at any time. In accordance with ethical regulations, parental consent was obtained for those under the legal age for health consent (16 years in Norway). Due to the sensitivity of the topics discussed during the interviews, relevant referrals or subsequent follow-ups were arranged by a clinical psychologist and project manager if necessary. All the participants received a follow-up call within two weeks after the interview to assess their need for referral to a clinical psychologist and obtain their feedback regarding how they experienced being interviewed about the topics in question. Three participants wished to receive follow-ups after the interviews and were referred to a clinical psychologist.

## Findings

In this study, we examined young people in families with HD’s experiences of being supported by others outside the parent–child relationship. The participants’ accounts differed in many ways, including when their parents developed symptoms, the severity of the disease, and the disease’s impact on them. Despite these differences, they still seemed to share similar descriptions of how the support of others had helped them cope with and adapt to stressors, as well as what barriers they experienced. Their experiences are presented within two superordinate themes, *‘How support helped’* and *‘Support barriers’*. Table 3 summarises the superordinate themes and corresponding main themes and subthemes.

**Table 3.** Superordinate themes and corresponding main themes and subthemes.

Superordinate themes	Main themes	Subthemes
How support helped	I felt connected to someone	I felt loved and cared for
		I felt less alone
	I learned how to cope	I developed skills
		I developed an understanding
	I got a break from reality	I had an escape
		My responsibilities were reduced
Support barriers	My needs for support were not understood	Parents are gatekeepers to support
		Others lack of knowledge about my situation
	I did not know if involving others would help	Protecting my family
		Protecting myself

## How support helped

The first superordinate theme, ‘*How support helped,’* reflects the different ways others had supported our participants in coping with and adapting to the variety of stressors they experienced during childhood and adolescence. While some participants described having dynamic networks of family, friends, or professionals that had been or could be a form of support when needed, this seemed not to be the case for most participants. Instead, the presence of one significant person or group were the ones providing support. Three main themes were derived reflecting the different ways in which relationships with others were described as supportive: ‘*I felt connected to someone,’ ‘I learned how to cope,’* and *‘I got a break from reality.’*

### I felt connected to someone

Feeling connected to others was an important source of support that seemed to reflect two emotional functions: ‘I *felt loved and cared for’* and ‘*I felt less alone*’.

**I felt loved and cared for:** Relationships had helped participants when these connections made the young people feel loved and cared for. These were relationships that subtly and continuously helped in the background, such as displaying love and care by staying close and being involved in minor or major events that occur on an everyday basis:

I have always been very connected to my grandfather. If he had not been there, I wouldn’t be … here … We didn’t talk all that much, but at the same time, I always knew he was there and that he was my protector … . He showed me that he loved me just by being there and that I had someone who cared. It was probably what I had been missing the most, but I had him … who made me who I am and gave me the strength to pull through the way I did. (Tina; adult female, father with HD)

Tina highlights several important functions of such supportive relationships. Through these relationships they had someone who they were close to, who worked to keep them safe and secure, who took notice of them and their situation, and who was on their side. Their home environments were more often than not characterised by chaos and conflict and parents who did not have the capacity to show their children the love and care they needed. In this sense, having close ties with others who made them feel loved and cared for provided a ‘buffer’ that helped participants cope with and adapt to the distressing emotions they had felt at home: ‘*My aunt was always the buffer I had. She could be there when my mom and dad were not there, and she would acknowledge me’* (Tammy; adult female, mother with HD).

**I felt less alone:** Relationships with others with similar or comparable experiences had also helped because this connection made the young people feel less alone. These ranged from short encounters to lifelong and profound relationships. One commonality among these relationships was that others with similar experiences seemed to convey an implicit understanding of the young people’s true experiences, which helped by making them feel less alone:

In the beginning, I felt all alone. I felt like I was the only one in the world who had these feelings and these experiences … But when I got to this camp for young people in families with HD, I was really happy to see that I was not the only one and to feel that, finally, I had someone I could talk to. I felt that everyone there was just like me and that I didn’t stick out. Everyone was the same and spoke about the same things. Everyone could share their feelings in the same way, and everyone would understand everything that was said. (Danielle; young adult female, father with HD)

As Danielle demonstrates, a mutual or shared understanding helped by providing a sense of belonging, companionship, and normalcy and compensated for or ‘buffered’ feelings of being alone that many had experienced in other relationships. A sense of trust was also established through these relationships, which provided the young people with the opportunity to have a confidante who could validate their feelings and concerns.

### I learned how to cope

Relationships had also consistently helped when they provided the young people with information or advice. The supportive ingredient of this help seemed to be the provision of different tools to cope: ‘*I developed skills*’ and ‘*I developed an understanding*’.

**I developed skills:** Some participants described how acquiring skills to cope with their feelings and reactions to stressors was helpful. Relationships with others were viewed as supportive when they helped to alter destructive thoughts and reactions to stress and develop strategies they could continue to use to work through problems:

What has helped is that we have someone who actually steps into our home on a day-to-day basis. She is here a few times a week and talks with my mom and dad, talks with me, talks with my brother. Mostly with me … It has helped because we almost always talk about how to handle the conflicts, and as you know, conflicts are one of those things that will intensify when someone in the family gets Huntington’s … . (Zachary; teenage male, father with HD)

As Zachary suggests, not all challenges related to HD can be eliminated. Instead, others help by teaching young people to develop tools via which to work around and adapt to obstacles. Different skills taught by support providers had strengthened the young people’s coping abilities. Other participants described how such tools gave them the self-efficacy necessary to handle ongoing feelings and concerns and communicate their thoughts and feelings to others using effective strategies.

**I developed an understanding:** Some participants’ had also found support in those who specifically increased their understanding of HD. Some talked about those who helped them broaden their perspectives on the disease and develop a positive outlook and hope, despite the possibility that they could also inherit the disease one day:

I have a lot of good experiences from the stays we had at these healthcare facilities where the whole family was included. I processed a lot there, changed how I viewed things. The more information I got, the safer I felt. In terms of the possibility that I could become ill one day too, I learned that everyone with HD is not the same. That was really good to know. (Dawn; adult female, mother with HD)

As Dawn highlights, information and understanding was a support because it helped the participants view negative experiences or thoughts about their futures in a more adaptive light. An increased understanding gave them room to process distressing experiences from the past and helped them prepare for the progression of their parents’ disease in the future with less distress.

### I got a break from reality

Relationships also consistently helped participants’ cope by providing assistance. Support that came with assistance reflected two types of relief from stress: ‘*I had an escape’ and ‘My responsibilities were reduced.’*

**I had an escape.** Several participants described how relationships were supportive because these relationships provided a sense of distance or escape from the distress the participants were experiencing at home. For some, these relationships were with their friends and family, who provided them with the benefit of having somewhere to be outside their home. Dawn highlighted how organised activities had been of help to her:

I did sports, played instruments, did art, and went hiking. I was given time to be just me but also to get out and to have someone who would see that I was good at something. It was a little bit of a kick, doing sports and activities and having someone notice … . Someone who would acknowledge me during those years. (Dawn; adult female, mother with HD).

In this context, the actions and behaviours of others were helpful because they provided a direct sense of distance from home and an emotional distraction from distressing experiences. Through the eyes of someone outside their family, the young people could also have a break from their distressing thoughts and feelings and build self-esteem, a sense of self, and friendships without constant thoughts about the disease.

**My responsibilities were reduced:** Relationships also helped when others’ actions lessened the overwhelming responsibilities young people felt at home. In contrast to the previous sub-theme reflecting the supportive element of relationships that made young people feeling like they had somewhere to escape, these experiences reflected tangible measures others provided at home and how they helped. For some, support providers alleviated growing responsibilities by providing separate housing for the young people and their parents who could not live at home, or by taking over care tasks such as cleaning the house or personal care for their parent with a disease. For other participants, the measures employed by support providers did not alleviate participants’ responsibilities, but they were helpful because they changed their sense of responsibility for the better:

After we got a puppy, everything changed, or at least a lot. It was like therapy because the dog and my dad are best friends. The dog quickly learned that we need to look out for Dad. He will notice everything and is really good at it … . When we are somewhere else in the house, the dog will come running and bark to let us know when there is something wrong with Dad and we need to come and help … . (Danielle; young adult female, father with HD)

Although the support provided to this participant came with fur and four legs, Danielle’s account still highlights the importance of support that changed young people’s sense of responsibility. Because young people often worry about and feel responsible for the safety and care of their parents, measures that help them care for their parents also decrease the burden of responsibility, resulting in their home lives feeling less overwhelming.

## Why support was hindered

While reflecting on how others had supported them during their childhood and adolescence, participants generally placed more emphasis on how they felt they had lacked support from others and the many barriers to support they experienced. The second superordinate theme, *Why support was hindered,* therefore covers the participants’ descriptions of obstacles to social support, illustrated by two themes: ‘*My needs for support were not understood’* and ‘*I did not know if involving others would help’,* and reflects barriers that had the potential to hinder all ‘types’ of support for our participants.

### My needs for support were not understood

A general lack of understanding of HD, how the disease truly affected their lives and their needs was described as a major barrier to receiving support. These experiences were captured by two sub-themes; ‘*Parents are gatekeepers to support’* and ‘*Others lack of knowledge about my situation’*.

**Parents are gatekeepers to support:** Almost all of the participants described how their parent with HD’s lack of insight and awareness of their disease was a barrier to receiving support: ‘*It’s the most difficult part about this whole thing when you are a young carer who wants help but you are not getting anywhere because your parent is denying that they have a disease’* (Ian; young adult male, mother with HD). Some parents were described as unaware of how their own disease developed and oblivious to or in denial about the changes and challenges it was creating in their children’s lives. In many instances, their caregivers with the disease were also the gatekeepers regarding whether others could gain insight into the true nature of their home lives and thus make the changes needed for the children. However, because their caregivers’ disease often progressed without being noticed by others outside the home, obtaining help and support was difficult. For instance, when in contact with others, parents could disguise symptoms, often creating an illusion that everything was all right. In addition, some consequently refused or ignored any help or medical attention because they did not find it necessary:

She did not understand that I needed someone to talk to or why someone came from the hospital to inform us and our friends about what this disease is … . Even to this day, she claims that she is not sick … . (Morgan, teenage female, mother with HD)

Numerous descriptions similar to morgan’s account further highlight that the need for support went undetected by others and conflicts between the needs of young people and the needs of parents with HD were generally resolved in favour of the parents.

**Others lack of knowledge about my situation:** When others lacked knowledge about the true nature of HD, this created a range of obstacles that prevented young people from finding support. For some, this meant that their need for support went undetected by potential support providers. This played into parents’ denial of the disease because the perception of the needs of the young people was often based on the information provided by parents with HD. As a result, their children’s need for support was not taken seriously. Some participants highlighted the fact that this meant they were not provided with continuous support, as is necessary considering the progressive nature of the disease. Others brought forth the idea that the need for mandatory or automatic support systems and measures was not recognised, as it normally would have been for children whose parents had illness or disease with better understood support needs. A general lack of knowledge also seemed to leave others not knowing what to do to help the young people:

I stood all alone as a teenager … . I later realized that others knew more than they let on, and I can’t really understand why they didn’t go in to check if things were working at all. Perhaps, they didn’t know how to tackle it. Perhaps, it was just easier to do nothing when they didn’t know what to do. (Lori; adult female, mother with HD)

As this Lori suggests, believing that they did not have the appropriate tools to help others, potential support providers shied away from intervening in their lives. These experiences could also be related to the stigma, misconceptions, and fear attached to the disease, leaving young people isolated from potential support.

When others lacked knowledge about HD, the support that was actually provided often did not align with young people’s support needs:

I don’t think they understand that they don’t have the full picture and that I don’t feel like I can talk to them at all. Even though I know they are only trying to be supportive [and] they want me to talk to them and want to help me, it’s like … it only makes it worse. (Brooke; teenage female, mother with HD)

As Brooke suggests, when potential support providers did not understand the true nature of the young peoples’ situation, any support received could feel wrong or misplaced and, in turn, increase young people’s distress instead of decreasing it. Generally, meeting others who did not understand seemed to come at a great cost; these situations caused them to feel rejected and helpless in trying to find someone to turn to, potentially deterring them from seeking the help and support of others in the future.

### I did not know if involving others would help

Relying on others outside the parent–child relationship for support would, in many instances, also mean allowing others access to their families, home lives or personal thoughts and feelings. Fear of the consequences of this exposure led young people or other family members to avoid the potential support of others, as captured by two sub-themes: ‘*Protecting my family’* and ‘*Protecting myself*’.

**Protecting my family:** When young people or other family members were scared of the potential repercussions that could come with others gaining insight into their families, support was hindered. Some young people attempted to protect their families from exposure by hiding their need for support or not reaching out to others. The young people had considered several reasons to do so. Some were scared that others would disrupt their family cohesion, such as separating them from their parent(s) or siblings, or that the help of others would trigger adverse reactions in their parents. Others expressed how they had felt guilty knowing that their need for support would be in conflict with the needs of their parents with HD; for instance, they feared that their parents or themselves would have to leave the home and wondered who would take care of them if the family did not:

When I look back on the situation today, I think it would have been a very good thing if I had allowed someone to see and take me away, but with children and loyalty, I don’t know if that would have worked … (Steven; adult male, father with HD)

As Steven demonstrates, some participants felt an innate loyalty to and need to take care of and protect their parents, even at the cost of finding help themselves. Other family members need to protect their families and themselves could also result in them rejecting and overlooking young people’s support needs. Many of the extended families of our participants had been overwhelmed by the responsibilities that have followed generations with HD. These social contacts, which could offer support because they were familiar with the young people’s situation and had had similar experiences themselves, had therefore, instead, been caught up in conflicting responsibilities, loyalties, and desires to care for those who had developed the disease, and they protected their own agendas over the support needs of the young people: ‘*Everybody had enough on their plate already, and they probably wanted to keep the issues within the four walls of our house’* (Steven; adult male, father with HD). Consequently, when it came to social support from extended family members, young people could be left without any neutral party who was acting in these young people’s best interest.

**Protecting myself:** When young people were too overwhelmed by the issues they were facing, this could become a barrier to receiving support. Getting help could also mean that the young people would have to give others access to their distressing personal experiences and be forced to address difficult feelings and topics that not all have the strength to face:

It’s not like this ‘no one sees me’ thing. I have plenty of people who have tried to follow up, tried to provide support. I have been bombarded with counselling sessions, so that’s not the problem. I just haven’t wanted to deal with it, haven’t been able to handle it. (Zachary; teenage male, father with HD)

As Zachary describes, some participants had avoided the help of others or did not reach out to others out of a sense of self-preservation or attempts to cope with ongoing and overwhelming stressful experiences to the best of their abilities. Accepting help or support from others could also mean giving others access to their lives and being exposed to potentially negative judgements or different treatment as a result:

I didn’t want them to treat me differently. I just wanted to be the same as everyone else. I felt they treated me like, ‘Oh, we have to take very good care and watch out for this one,’ and they didn’t do that with the other children, right? (Danielle; young adult female, father with HD)

As Danielle highlights, accepting support could jeopardise participants’ need to feel or appear normal. This fear consequently hindered or limited the help and support participants received from close friends, as young people often feared embarrassment or exposure due to bringing someone else into their lives.

## Discussion

This study aimed to investigate how relationships outside the parent–child context help young people who grow up with a parent with HD and explain why this group may not feel that they have the supportive relationships they need. The findings presents a number of new insights into the multifaceted and important roles others outside the parent–child context have in helping young people in families with high levels of stress and adversity, such as families affected by HD. The findings also detail the many barriers that must be overcome for this vulnerable group to receive the support they need.

Past research has shown the distressing circumstances young people in families with HD may endure, as well as showing that support from their caregivers can be severely compromised (e.g. Forrest Keenan et al., 2007; Kavanaugh et al., 2015; Kjoelaas et al., 2021; Vamos et al., 2007; van der Meer et al., 2012). The first main finding in this study builds on this knowledge by identifying the many important roles others’ or groups’ outside the parent–child context can serve in helping young people cope with and adapt to stressful life circumstances. On a general level, these findings mirror past theory and research by showing that relationships that help young people in families with high levels of stress and adversity can come in many forms, ranging from formal contacts such as healthcare professionals and teachers, to informal relationships, including relatives, neighbours, peers, and even devoted pets (Taylor, 2011). As with past research specifically examining the support needs of young people in families with HD regarding their caregiving duties (Kavanaugh et al., 2015), our participants had been helped through various types social support, including emotional, informational, and instrumental support. However, because research on the role social support plays under stressful circumstances often fails to explain how these relationships actually help someone (Thoits, 2011), our findings are unique due to the in-depth knowledge they provide.

First, we found that emotional support helped young people in families with HD by creating connections that made them feel loved, cared for, and less alone. It is theorised that one way social support can help with stress is when relationship indirectly reduce or ‘buffer’ the physical and emotional arousal related to stressors, helping sustain someone’s positive and adaptive emotions. This is referred to as ‘emotional sustenance’ (Thoits, 2011). This seems applicable to how participants in this study described the helpful elements of emotional support. For our participants, relationships seemed to have the capacity to significantly help them cope and adapt through the sheer connectedness and value others had transferred to them. As demonstrated by this and other studies, young people with a parent with HD can experience enduring emotional strain and feelings of isolation and loneliness, and at the same time have caregiving relationships where emotional availability is compromised (e.g. Forrest Keenan et al., 2007; Vamos et al., 2007), finding ways to strengthen or build connections that provide these types of emotional support may be particularly important. Our findings suggest that emotional support from others outside the parent–child context can protect young people by sustaining positive or adaptive feelings, such as safety, and by reducing and buffering emotional distress related to a lack of love, a lack of security, or feelings of loneliness. Support providers are encouraged to help young people in families with HD strengthen or build relationships that provide these types of emotional support, such as close family members or others with similar experiences. For instance, young people in families with HD can easily be connected to available arenas for peer support, such as the Huntington’s Disease Youth Organisation (HDYO), Young People Affected by HD (YPAHD), and the Huntington’s Disease Society of America’s National Youth Alliance (NYA).

Second, in addition to relationships that sustained positive emotions through adversity and distress, our participants had also been helped by relationships that actively changed the way they handled stressors and distressing emotions. These types of changes seemed to stem from informational support. The way our participants described informational support seems to reflect what is theorised to be stress buffering by reducing someone’s psychological or physiological stress-related arousal, which is called ‘active coping assistance’ (Thoits, 2011). For our participants, relationships had helped because they taught them the skills needed to handle ongoing issues, which helped them think about, approach, and tackle stressors. In turn, they developed skills such as self-efficacy and effective communication that buffered or reduced emotional distress through building the capacity to handle ongoing challenges. Past research has also focused on the importance of coping strategies when families are affected by HD (Ciriegio et al., 2020; Forrest Keenan et al., 2007). Because studies have shown that these young people often lack information and communication about the disease in their caregiving relationships (e.g. Forrest Keenan et al., 2003; Stuttgen, McCague, Bollinger, Dvoskin, & Mathews, 2021), finding ways to strengthen or build relationships that they can rely on for information and help to understand their situation and HD may be particularly important. Our findings suggest that relationships outside the parent–child context can play a central role in providing information and teaching coping strategies that can help young people in families with HD cope and adapt. Support providers, such as family members, genetic counsellors, or other health care workers are encouraged to ensure that young people have relationships that give this type of informational support, such as providing help to re-structure unhelpful thoughts and worries about their own risk of having HD and whether or not to get tested.

Relationships also helped by providing instrumental support that actively changed the way the participant’s handled stressors by reducing or alleviating the many stressors they encountered at home. Building and strengthening instrumental support may be particularly important for this group because they are often overwhelmed by responsibilities at home and caregiving tasks and may find only limited time for social activities (e.g. Kavanaugh, 2014; Lewit-Mendes et al., 2018). According to our findings, instrumental support can help by transforming young people’s feelings of being overwhelmed and can distract them from distressing emotions. In turn, these types of support can also build self-esteem, close connections, and identities that will help protect these young people against current and future stressors. Support providers are therefore encouraged to provide this type of instrumental support. Such support can include financial and practical aid to the families or the young people and appropriate help and support to the parent with HD to alleviate some of the responsibilities and caregiving tasks they could be left with.

The second main finding in this study was the many barriers that hindered the participant’s to feel supported. On the one hand barriers were created by a lack of acknowledgement of the young people’s situation or a lack of recognition of their needs. On the other hand, they related to the way in which young people and their family members themselves did not allow others to see their needs for support. On a general level, these findings are in line with past research indicating that children in families with HD feel as if they have limited opportunities to find the necessary support (e.g. Kavanaugh et al., 2015; Lewit-Mendes et al., 2018). Other studies have also found that high levels of social support barriers is related to poor mental health trajectories after adversities (e.g. Arnberg, Hultman, Michel, & Lundin, 2013; Thoresen et al., 2014). Importantly, in these studies, experiences of being hindered in obtaining social support were highly associated with psychological distress.

Many of the barriers young people with a parent with HD experienced were related to the nature of their parents’ disease and the fact that those who could have helped did not have the necessary knowledge to interpret the young people’s support needs correctly. The development of symptoms associated with HD, such as a lack of self-insight and symptom awareness, had severely complicated young people’s access to the necessary support. While this is one of the first studies to provide an in-depth understanding of the many ways symptoms of HD can hinder social support within affected families, the neuropsychological and neurocognitive symptoms creating these barriers have been well documented (McColgan & Tabrizi, 2018). Even in the early phases of HD, before the development of visible motor disturbances and a set diagnosis, HD has been found to create fundamental challenges to someone’s ability to understand his or her own and other’s mental states and needs, as well as to create deficits in the ability to identify inappropriate behaviours (Eddy & Rickards, 2015). The results of our study clearly demonstrates how the early and developing cognitive symptoms of HD may not only affect the individual with the disease, but also, when left undetected, severely limit their children’s access to support. For support providers it is important to be aware of these barriers and address them directly.

As is evident in our findings, the heritability of diseases such as HD also means that entire families and generations can be indirectly affected. In this study, this could severely compromise relatives’ provision of support for young people. While extended family members normally have the potential to be of great support, their fear, loyalty, and protectiveness could also lead them to prioritise the affected parent’s needs above the child’s. According to the social support theory literature, close family ties may be the most accessible and important sources of support because of their closeness to the distressed individual (Thoits, 2011). However, past research has suggested that family norms of secrecy or fear of exposure follow many families with a history of HD, making it difficult for young people to gain support from these relationships (Forrest Keenan et al., 2015). As such, family support can be compromised because they can be ‘too close to the situation’ to be of help. Because they are often upset about the same situations or behaviours, relatives may minimise the child’s experiences, distance themselves from the issues, attempt to solve the problem as quickly as possible, or force a positive outlook that does not actually address a young person’s true needs (Thoits, 2011; Thompson et al., 2015). Therefore, support providers are encouraged to also connect young people to arenas for social support outside their families.

Our findings also suggest that, because many of the symptoms of HD reflect impairments in thinking and are not directly observable, young people’s needs for support can easily go unnoticed or be misunderstood if helpers lack knowledge about the disease and its consequences on young people. Social support literature also suggests that helpers can be ‘too distant or unfamiliar’ with an issue to effectively provide support (Thoits, 2011). For our participants, this had come at a great cost, leaving many without the necessary support throughout their upbringing. The general lack of knowledge young people experienced as a barrier had three main consequences. First, when others lacked knowledge about and an understanding of the distress young people were coping with at home, children’s support needs could easily be neglected. For instance, the needs of the parent with a disease may be addressed, while the needs of their children are not. Second, when others lacked knowledge about the young people’s situation, provided support was not in line with the children’s true needs. In line with research that also suggesting that support can yield a range of negative outcomes (Maisel & Gable, 2009), wrong types of support ended up exacerbating instead of reducing stress. Third, when others lacked knowledge, they did not know what to do in order to help, and some participants had even experienced rejection when reaching out for support. Feeling rejected or feeling that the support they receive is negative is perhaps particularly damaging to vulnerable groups. Such experiences may cause someone to feel resentment and betrayal, and seriously compromise their ideas about available support (Thoits, 2011). In turn, as suggested by research on groups with other severely distressing or traumatising experiences, negative social constraints that comes with support is associated with mental health difficulties, and can lead a person to not utilise potential support that might otherwise be within reach, creating a sense of learned helplessness (Andrews, Brewin, & Rose, 2003; Kaniasty & Norris, 2008). Based on these findings, we encourage future studies to investigate ways to improve the public’s understanding of the impact HD has on families.

This seems, at least in part, to be reflected through the barriers described in which the young people themselves had hindered support. In some instances, the young people were so overwhelmed by their situation that they did not have the capacity to receive or reach out for support. Social support literature also suggests that experiences can be so devastating and upsetting that supporters themselves are emotionally overwhelmed and become avoidant to protect themselves from distress (Thoits, 2011). Those in contact with these young people are therefore encouraged to provide support that accounts for how distressing experiences affect someone at an individual level. Other obstacles may include young people’s feelings of responsibility to take care of their parents, as well as the loyalty they feel. The idea that children can experience internal conflict resulting from loyalty and protectiveness is not unique to those growing up with a parent with HD. In fact, young carers with a parent with chronic illness frequently feel the need to provide help and protect both parents, and importantly, suppress their own needs to do so (Chikhradze et al., 2017). Nonetheless, young carers also frequently report positive effects, including early maturity, close relationships to their parents, and a preparedness for life (Chikhradze et al., 2017). Support providers are encouraged to help by promoting healthy aspects of having responsibilities and at the same time hindering potentially harmful costs of caring. This includes talking with young people about their needs for support and being aware that family members might have insecurities about the possible risks that can come with receiving support. In adolescence, this strong alliance with parents tends to shift, and having relationships with peers and feeling normal become more important. The literature on social support suggests that some recipients of support can have negative reactions when the support is ‘too visible’; adolescents in particular may not want to draw attention to the problem, feel as though they owe others, feel controlled or feel devalued (Maisel & Gable, 2009; Thoits, 2011). This may reflect why the young people in this study frequently described what appeared to be a paradox in terms of support, both needing and hindering the support of peers.

### Strengths and limitations

This study included participants who reflected on both current and past experiences from their childhood and adolescence. This means that actual support, perhaps particularly support provided by healthcare services, do not reflect the forms of support available today. However, despite the many differences in the availability of support, participants’ descriptions of factors defining whether support was experienced or missed did not seem to vary across time, as one might expect. Therefore, we believe that our findings demonstrate the core elements of support which can be applied across settings, times, and places. Another limitation could be the nature of the sampling. For instance, it is possible that those who grew up with a parent with HD who wished to be a part of this study had more difficult experiences that they wanted to share, whereas those with few negative experiences may not have felt the same need to participate. While a major strength of the qualitative approach in this study is that it provides a greater depth of understanding of participant’s experiences than other methods, the findings should still be considered transferable rather than generalisable.

## Conclusion

Past research has shown that young people in families with HD may experience enduring adversity and distress, combined with potentially compromised parent–child relationships that in other contexts provides support. We therefore examined how relationships with others outside this context could help this vulnerable group cope and adjust, as well as the barriers they encountered to feel supported by others. The findings highlight the important role of building and strengthening relationships with others outside the parent–child context, as well as the ways in which these relationships can buffer the many stressors this group of young people face. Supportive relationships were found to help young people by sustaining positive and adaptive emotions through connections that provide love, care, and belongingness and to handle distressing emotions by teaching coping skills and relieving or alleviating stressors at home. The many barriers to support these young people had encountered also seemed to hinder their abilities to cope with and adjustto the challenges that came with the disease. Barriers included the fact that their need for support was not recognised and understood by their parent with HD and that others who could provide support lacked the knowledge needed to understand the nature of the challenges these young people were facing. Support was also hindered by avoidance and a need to protect themselves and their families from intervention and possible harm. Together these findingssuggests that support may not be experienced as helpful unless it is perceived as addressing the issues young people themselves feel they are facing. Therefore, those in contact with families with HD need to gain knowledge and understanding of the disease and young people’s needs, thus helping them overcome the many obstacles they face.
